# *Dendrobium officinale* polysaccharide ameliorates high-fat diet-induced hepatic lipid metabolic disorder via the SIRT6/PGC-1α signaling axis

**DOI:** 10.3389/fnut.2026.1763341

**Published:** 2026-06-02

**Authors:** Binhong Hu, Ling Yao, Xin Deng, Wei Zhao, Kuo Chen, Jiacheng Zhang, Zhiqiang Li, Jin-ping Li, Wei Jiang, Huabao Liu

**Affiliations:** 1 College of Traditional Chinese Medicine, Chongqing University of Chinese Medicine, Chongqing, China; 2 College of Chemistry and Life Sciences, Chengdu Normal University, Chengdu, Sichuan, China; 3 Department of Medical Biochemistry and Microbiology, Uppsala University, Uppsala, Sweden; 4 The First Affiliated Hospital of Chongqing University of Chinese Medicine, Chongqing University of Chinese Medicine, Chongqing, China; 5 Department of Animal Science, Fujian Agriculture and Forestry University, Fuzhou, Fujian, China; 6 Department of Molecular and Cell Biology, Far Eastern Federal University, Vladivostok, Russia; 7 The First Affiliated Hospital of Zhengzhou University, Zhengzhou, China; 8 Sichuan Provincial Key Laboratory for Development and Utilization of Characteristic Horticultural Biological Resources, Chengdu Normal University, Chengdu, Sichuan, China

**Keywords:** *Dendrobium officinale* polysaccharide, gut microbiota, HFD, lipid metabolism, PGC-1α, SIRT6

## Abstract

**Aims:**

This study aims to explore the potential therapeutic effect of *Dendrobium officinale* polysaccharide (DOP) on non-alcoholic fatty liver disease (NAFLD) induced by high-fat diet (HFD), and to elucidate the underlying mechanism involving the SIRT6/PGC-1α signaling axis and the regulation of the gut microbiota.

**Methods:**

We extracted and characterized DOP. We established a rat model of NAFLD induced by HFD and evaluated the efficacy of DOP by integrating multi-omics techniques (transcriptomics, metabolomics) and 16S rRNA sequencing. To verify the specific role of SIRT6, we introduced the SIRT6 inhibitor OSS_128167 in the primary hepatocyte model induced by oleic acid/palmitic acid (OA/PA).

**Results:**

DOP significantly alleviated liver steatosis, oxidative stress, and lipid metabolism disorders induced by HFD. Multi-omics analysis indicated that DOP regulated liver glycerophospholipid metabolism and restored intestinal microbiota homeostasis, significantly increasing the abundance of beneficial bacteria such as *Lactobacillus*. Mechanistically, DOP activated the liver SIRT6/PGC-1α signaling axis, thereby enhancing antioxidant defense and inhibiting lipogenesis. Crucially, *in vitro* experiments confirmed that the SIRT6 inhibitor OSS_128167 eliminated the protective effect of DOP on lipid accumulation, confirming that the effect of DOP depends on SIRT6.

**Conclusion:**

DOP improves NAFLD through dual mechanisms of regulating the gut-liver axis homeostasis and directly activating the liver SIRT6/PGC-1α signaling pathway. The results of this study provide a theoretical basis for developing DOP as a drug for the treatment of NAFLD.

## Graphical Abstract

**Figure:**
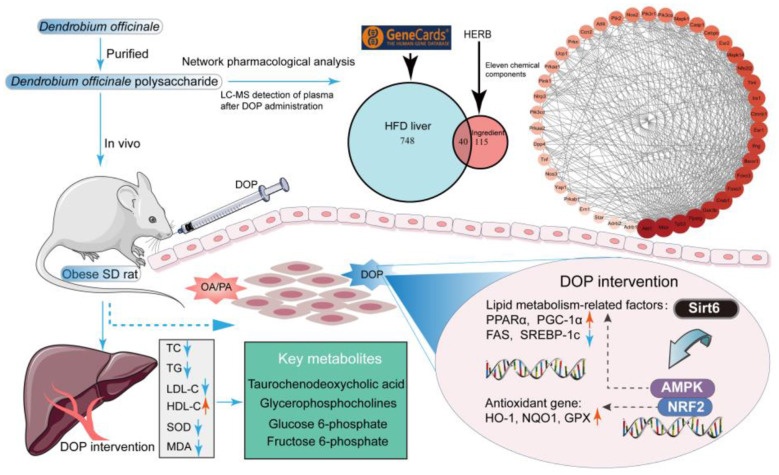

## Highlights

- DOP alleviates HFD-induced metabolic disorders via the SIRT6/PGC-1α axis.
- Multi-omics shows DOP rectifies gut microbiota dysbiosis and hepatic profile.
- DOP activates NRF2, upregulates PPARα, and suppresses SREBP-1c.
- SIRT6 inhibition abolishes DOP's hepatoprotection, confirming its core role.

## 1 Introduction

High-fat diet (HFD) is a major driving factor in the onset and progression of metabolic syndrome and non-alcoholic fatty liver disease (NAFLD), which are characterized by hepatic lipid metabolic disorder, enhanced oxidative stress and gut microbiota dysbiosis (1–3). Accumulating evidence indicates that HFD-induced disruption of gut microbial composition leads to abnormal regulation of energy metabolism and immune function, thereby promoting hepatic lipid accumulation and oxidative injury, ultimately accelerating NAFLD progression (3, 4). Currently no approved targeted therapies for NAFLD are available, posing a major challenge for its clinical management. First-line approaches, including lifestyle interventions and limited drug options (e.g., lipogenesis modulators and vitamin E), often fall short due to limited efficacy or potential side effects, failing to meet long-term treatment needs (5). This unmet clinical need has driven research efforts toward developing novel pleiotropic agents that can concurrently target multiple pathological pathways, such as hepatic metabolism, oxidative stress as well as gut microbiota dysbiosis.

In the search for new multi-target drugs, traditional medical systems offer valuable clues. *Dendrobium officinale*, a rare medicinal herb in traditional Chinese medicine, is used for “nourishing yin, clearing heat and promoting body fluid” (6, 7). Natural polysaccharides existing in plants and other organisms are attracting attention to explore their potentials for developing therapeutic agents, due to their structural diversity and broad biological activities (8–10). *Zexie Tang* polysaccharides and *Stevia rebaudiana* root polysaccharides can improve NAFLD induced by HFD by regulating metabolism and modulating intestinal microflora (11, 12). Among them, DOP have been reported having antioxidant, lipid-lowering, and gut microbiota-modulating properties, as well as hepatoprotective effects in metabolic disease models (13–18). However, the core molecular mechanisms underlying the observed effects of DOP on lipid metabolism remain largely unclear.

Sirtuin 6 (SIRT6) is a critical regulator of energy metabolism and oxidative stress. It maintains hepatic homeostasis by inhibiting glycolysis, promoting mitochondrial function, and activating antioxidant signaling (19–22). Its downstream transcription factor, nuclear factor erythroid 2–related factor 2 (NRF2), induces the expression of antioxidant genes, alleviates lipid deposition, and reduces oxidative damage (23–25). Moreover, SIRT6 can coordinate energy metabolism and antioxidant responses through regulation of PGC-1α and its interaction with PPARs and NRF2 (26–29). Recent evidence suggests that the SIRT6/PGC-1α signaling axis plays a protective role against HFD-induced metabolic disturbances, indicating a promising target for management of NAFLD as well as related metabolic disorder (20).

To find out the mechanisms of DOP in modulating high fat induced liver damage, this study employed an integrated strategy combining network pharmacology, 16S rRNA sequencing, transcriptomics, and metabolomics to evaluate the effects of DOP, with the aim to elucidate its regulatory effects on hepatic metabolism and gut microbiota changes in HFD-fed rats. We specifically focused on the question whether the hepatoprotective effects of DOP are mediated through the SIRT6/PGC-1α signaling axis. The results illustrated the key pathways by which DOP alleviates HFD-induced metabolic dysfunction, providing a theoretical basis for its development as a potential functional food supplements or therapeutic agent.

## 2 Materials and methods

### 2.1 Reagents

*Dendrobium officinale* kimura & Migo was obtained from Fengshang Biotechnology Co., LTD. (Sichuan, China), and while used the voucher specimen for storage (No. 2021041678DO). The extraction and characterization of DOP are detailed in Supplementary Material 1. Enzyme-linked immunosorbent assay (ELISA) kits for superoxide dismutase (SOD), malondialdehyde (MDA) were procured from Hepeng Biotechnology Co., LTD. (Shanghai, China). Triglyceride (TG), total cholesterol (TC), low density lipoprotein cholesterol (LDL-C), and high-density lipoprotein cholesterol (HDL-C) were purchased from Nanjing Jiancheng Bioengineering Institute (Nanjing, China). Additionally, SIRT6 inhibitor, OSS_128167 purchased from McLean (Shanghai, China). All antibodies targeting SIRT6, NRF2, HO-1, NQO1, GPX, p-AMPK, AMPK, PGC-1α, and SREBP-1c were acquired from Abcam (Shanghai, China). PPARα antibody was acquired from Abclonal (Wuhan, China). FAS and β-actin were acquired from Cell Signaling Technology.

### 2.2 Network pharmacology

In the Herb database, obtain the target genes corresponding to the 11 components in the plasma of rats fed with DOP. From the GeneCards database, collect the liver-related targets of HFD. The Venn diagram shows that there are 40 intersection targets between the component targets and the diseases. Continue to use these 40 targets for PPI, GO, and KEGG analysis. Import the intersection targets of component and disease into the “Multiple proteins” section of the STRING database. Then, select “Rattus norvegicus” under “Organisms” for protein-protein interaction (PPI) analysis. Set the minimum required interaction score in “Settings” to 0.4, and select to hide the disconnected nodes in the network. Set the number of clusters in “Clusters” to 3 to obtain the PPI network. Import the data file into the Cytoscape 3.10.1 software. Through degree centrality (degree) screening, select data with a value greater than the threshold to obtain the core network diagram of the target. Import the intersection targets of component and disease into the DAVID database. Use “Shortcut to DAVID Tools” and “Functional Annotation” in the “DAVID Tools” to conduct GO (Gene Ontology) biological process (BP), cellular component (CC), and molecular function (MF) enrichment analysis as well as KEGG signaling pathway analysis. Export the results and filter the data with *P* < 0.05,. Make the top 20 of KEGG into an enrichment bubble chart, and the top 10 of GO into BP, CC, and MF colored enrichment bar charts. Visualize the enrichment analysis results.

### 2.3 Molecular docking

The 2D structures of small-molecule ligands were retrieved from the PubChem database (http://pubchem.ncbi.nlm.nih.gov/) and converted into 3D structures using ChemOffice software, which were then saved as mol2 files. Protein targets were obtained from the RCSB Protein Data Bank (http://www.rcsb.org/), and high-resolution crystal structures were selected as docking receptors. Protein preprocessing, including removal of water molecules and phosphate groups, was performed using PyMOL 2.6, and the processed structures were saved as PDB files. AutoDock 1.5.6 (30) was used to prepare both protein and ligand structures by adding hydrogen atoms, removing water molecules, and assigning torsional degrees of freedom for the ligands. Docking grid box coordinates were then defined. Molecular docking was carried out using AutoDock Vina to evaluate protein–ligand interactions. The optimal binding conformation was determined based on docking scores. The docking results were further visualized using Discovery Studio 2019 and PyMOL 2.6 to generate 2D interaction diagrams and 3D structural analyses of compound–residue interactions.

### 2.4 Molecular dynamics simulation

Molecular dynamics (MD) simulations were performed using GROMACS 2022 (31). Force field parameters were generated using the pdb2gmx tool in GROMACS and AutoFF web server. The AMBER14SB force field was applied to the receptor protein, while the GAFF2 force field was used for ligands. The systems were solvated in a cubic TIP3P water box with a 1 nm buffer and counter-ions were added using the gmx genion tool to maintain electrostatic neutrality. Long-range electrostatic interactions were handled with the Particle Mesh Ewald (PME) method, and a cutoff distance of 1 nm was applied. Bond constraints were treated with the SHAKE algorithm, and the Verlet leapfrog algorithm was employed with a 1 fs integration time step. Prior to MD simulations, systems were energy-minimized through 3,000 steps of steepest descent followed by 2,000 steps of conjugate gradient minimization. The minimization procedure included: (i) constraining solutes with energy minimization of water molecules; (ii) constraining counter-ions with energy minimization; and (iii) unconstrained energy minimization of the whole system. MD simulations were conducted under NPT ensemble conditions at 310 K and constant pressure for 100 ns. During the simulations, g-rmsd, g-rmsf, g-hbond, g-Rg, and g-sasa tools were employed to analyze root mean square deviation (RMSD), root mean square fluctuation (RMSF), hydrogen bonds, radius of gyration (Rg), solvent-accessible surface area (SASA), and Gibbs free energy, respectively.

### 2.5 Animal models

All animal experiments were approved by the Ethics Office of Chengdu Normal University, China (No. CNDU-20230412058R), and conducted in accordance with the ARRIVE guidelines and the National Institutes of Health Guidelines for the Care and Use of Laboratory Animals. A total of 48 healthy male Sprague-Dawley rats (100~150 g, 6 weeks old) (32) were randomly assigned into four groups after completing a 1week period of adaptive feeding: 1) CON: control group fed a normal diet; 2) HFD: high-fat diet (60% fat, 20% protein, and 20% carbohydrates) group (a continuous 8 week high-fat diet); 3) HFD-DOP: HFD group with DOP intervention (180 mg/kg (33); and 4) DOP: a group only treated with DOP (180 mg/kg) fed a normal diet. DOP (180 mg/kg) was daily given by gavage to the HFD-DOP and DOP groups after feeding HFD for 14 days. At the end of the 8 week experiment, all rats were fasted for 12 h and then euthanized using pentobarbital sodium. Blood samples were collected from tail veins and centrifuged at 1,000 g for 20 min at 4 °C to obtain serum. The liver tissue was collected postmortem, part of the tissue was fixed in 4% paraformaldehyde and part was stored at −80 °C until analysis. The blood was collected into two aliquots, serum was used for analysis of TC, TG, LDL-C, HDL-C, SOD, and MDA using a kit (Aladdin, China). Among them, for each group, samples randomly selected from 6 rats were used for the detection of biochemical indicators. Plasma was used for metabolite analysis. Intestinal contents were aseptically collected and snap-frozen in liquid nitrogen, and stored at −80 °C.

### 2.6 Histological and immunohistochemical analysis

The fixed liver tissues were paraffin-embedded, sectioned at 5 μm, and stained with hematoxylin–eosin (H&E) for histological evaluation. For immunohistochemistry, sections were treated with 3% H_2_O_2_, blocked with 3% BSA, and incubated with anti-Myeloperoxidase antibody (GB150145, Servicebio, 1:500), followed by HRP-conjugated goat anti-rabbit secondary antibody (GB23303, Servicebio, 1:200).

### 2.7 16S rRNA sequencing

Genomic DNA was extracted from the collected intestine using a fecal DNA extraction kit. The variable regions of the 16S rRNA gene (e.g., V3–V4) (34) were amplified, and Illumina sequencing adapters with sample-specific barcodes were added to the PCR primers for multiplexing. Purified amplicon libraries were quantified, pooled in equimolar concentrations, and sequenced on the Illumina MiSeq platform using a paired-end strategy. To obtain representative sequences, raw data were first processed with Cutadapt software for primer trimming. Quality control analysis was then conducted based on the default parameters of QIIME 2. Finally, the QIIME 2 package was used to identify representative reads for each amplicon sequence variant (ASV).

### 2.8 Analysis of metabolome in plasma and liver extracts

For extraction of metabolites from the liver, 30 mg of the frozen liver tissue was transferred to a 1.5 mL EP tube containing two small steel grinding balls and 400 μL of a 4:1 (v/v) methanol-water solution (containing L-2-chlorophenylalanine, 4 μg/mL). After pre-cooling for 2 min at −40 °C, the samples were homogenized in a grinding mill (60 Hz, 2 min), followed by ultrasound in an ice water bath for 10 min. The samples were then incubated again at −40 °C for 30 min before centrifugation at 13,000 g for 10 min. The resulting supernatant was collected and dried. The pellets were subsequently re-dissolved in 300 μL of a 1:4 (v/v) methanol: water solution. After swirling for 30 s, the samples were treated with ultrasound in an ice water bath for 3 min to facilitate dissolution. Finally, the samples were clarified by centrifugation at 13,000 g for 10 min, and the supernatants obtained were filtered through a 0.22 μm organic phase pinhole filter (Jinteng, Tianjin, China). All samples were then stored at −80 °C until further analysis. The metabolites in the plasma were extracted by precipitation of proteins using 4:1 (v/v) methanol-water solution (containing L-2-chlorophenylalanine, 4 μg/mL).

The metabolits in the samples (both liverand plasma) were analyzed by MS (Mass Spectrometry) following separation on an ACQUITY UPLC HSS T3 column (100 mm × 2.1 mm, 1.8 μm). Both positive and negative ion scanning modes were used to collect sample quality spectrum signals. Progenesis QI v3.0 software (Nonlinear Dynamics, Newcastle, UK) was used for baseline filtering, peak identification, integration, retention time correction, peak alignment, and normalization. Characterization was performed using The Human Metabolome Database (HMDB), Lipidmaps (v2.3), and METLIN databases, as well as EMDB2.0.

### 2.9 Transcriptome analysis

Total RNA was extracted from the frozen liver using TRIzol reagent. RNA purity and concentration were assessed using a NanoDrop 2000 spectrophotometer (Thermo Scientific, USA), and RNA integrity was evaluated with an Agilent 2100 Bioanalyzer (Agilent Technologies, Santa Clara, CA, USA). Transcriptome libraries were constructed using the VAHTS Universal V5 RNA-seq Library Prep Kit according to the manufacturer's instructions. The resulting libraries were sequenced on an Illumina Novaseq 6000 platform. The raw reads in FASTQ format were processed with fastp to remove low-quality reads, yielding clean reads for subsequent analysis. These clean reads were then aligned to the reference genome using HISAT2 (35). Gene expression levels were quantified as FPKM, and the read counts for each gene were obtained using HTSeq-count (36). Differential expression analysis was performed with the DESeq2 software package.

### 2.10 Cell culture and activity assay

Primary hepatocytes were isolated from normal rats (37). The cells were cultured in Dulbecco's Modified Eagle Medium (DMEM) supplemented with 10% fetal bovine serum (FBS; Sevier Biotechnology Co., Ltd.) and 1% penicillin-streptomycin (double antibiotic solution; Sevier Biotechnology Co., Ltd.). Upon reaching the logarithmic growth phase, cell suspensions were seeded into 96-well culture plates at a density of 5 × 103 cells per well (38). The plates were then incubated in a CO_2_ incubator at 37 °C for 12 h. To establish an *in vitro* model of liver lipotoxicity, the cells were co-treated with OA (oleic acid) at concentrations ranging from 0.2 to 1.4 mM for 24 h, along with PA (palmitic acid) at half the molar concentration of OA to optimize the induction conditions. After determining the optimal OA and PA concentrations, the cells were treated with various concentrations of DOP and incubated for an additional 24 h (16). Cell viability upon the induction and treatment with DOP was assessed using the Cell Counting Kit-8 (CCK-8) kit. Briefly, cells in 96-well culture plates were incubated for 4 h with 10 μL of CCK-8. The absorbance of each well was then measured at 450 nm using a plate reader. Cell viability was calculated according to the formula:


$$\begin{array}{r} Cell\;Viability\;Rate\;\left(\%\right)=\left(A_{\text{test}}-A_{\text{blank}}\right)/\left(A_{\text{control}}-A_{\text{blank}}\right)\\ \times \;100\%, \end{array}$$

where A_blank_ is the absorbance from wells containing culture medium and CCK-8 solution only (no cells), A_control_ is the absorbance from wells containing cells and CCK-8 solution, and A_test_ is the absorbance from test wells treated with DOP.

### 2.11 Real-time fluorescence quantitative PCR analysis (RT-qPCR)

The expression levels of sirtuin-6 (*SIRT6*), peroxisome proliferator-activated receptor gamma coactivator 1-alpha (*PGC-1*α), peroxisome proliferator-activated receptor alpha (*PPAR*α), fatty acid synthase (*FAS*) (relative to *GAPDH* levels) were estimated using fluorescent RT-qPCR. Cell cultures were prepared as described in section 2.10, and the cells were divided into five groups: a Control group (CON); a *SIRT6* inhibition group (SIRT6); an OA+PA model group (OA/PA); a liver lipotoxicity model + OSS (OA/PA+OSS); a DOP intervention OA/PA group (OA/PA+DOP); and a OA/PA + DOP + OSS (OSS_128167) (39). These cells were co-cultured with OSS_128167 at a final concentration of 100 μM for 24 h. The combined administration of OSS_128167 and DOP was carried out for 24 h as a joint intervention. Total RNA was isolated from the harvested cells using TRIzol reagent (TSP413). Cell mRNA was then reverse transcribed into cDNA using the Reverse Transcription Kit (Thermo Fisher Scientific, USA), according to the manufacturer's instructions. Fluorescent quantitative PCR was performed under the following conditions: an initial denaturation at 95 °C for 2 min, followed by 40 cycles of denaturation at 95 °C for 15 s, annealing at 60 °C for 30 s, and extension at 72 °C for 30 s. All primer sequences are listed in Table 1. Finally, the expression levels of the target genes were determined using the delta-delta Ct (2^−ΔΔ*Ct*^) method.

**Table 1:** Primers used for real-time fluorescence quantitative PCR analysis.

Name	Primer sequence (5' → 3')	Product size (bp)
*SIRT6*	F:5**'** GACTGGGAGGATGCGTTGCC 3' R:5**'** CGGTCATGTTTTGTGGGTTGC 3'	185
*PGC-1α*	F:5' CGCCTTCTTGCTCTTCCTTT 3' R:5**'** GCTGTCATACCTGGGCCTAC 3'	299
*PPARα*	F:5**'** ATCCACGAAGCCTACCTGAA 3' R:5' GGACCTCTGCCTCCTTGTTT 3'	184
*FAS*	F:5' ACCCGACTTCCTCTGGGATG 3' R:5' TGCTGAATACGACCACGCAC 3'	114
*GAPDH*	F:5**'** CCATCACTGCCACTCAGAAGA 3' R:5' ACATTGGGGGTAGGAACACG 3'	181

### 2.12 Western blotting analysis

Total protein was extracted from the primary hepatocytes cells and quantified using the bicinchoninic acid (BCA) protein detection kit (Nanjing Jiancheng, Nanjing). The lysates were then diluted with SDS-PAGE Loading buffer to a concentration of 2 μg/mL. Next, the protein lysates were separated by SDS-PAGE (on a 10% gel), and the resulting bands were transferred onto polyvinylidene difluoride (PVDF) membranes. The membranes were blocked with 5% skim milk powder for 1 h at 37 °C and were then incubated with primary antibodies overnight at 4 °C. After five washes (5 min each time) with TBS-T (Tris-buffered saline with 0.1% Tween^®^ 20 Detergent), the membranes were incubated with secondary antibodies at room temperature for 1 h. Finally, after further washes, the protein bands were detected by an ECL detection system. The bands were quantified using Image J software (NIH, Bethesda, Maryland, USA). All protein expression levels were normalized using β-actin as an internal control.

### 2.13 Statistical analysis

All statistical analyses were conducted using GraphPad Prism 9.5. One-way ANOVA followed by Tukey's multiple comparison test was used. *P* values were corrected for multiple comparisons. ^*^P < 0.05, ^**^P < 0.01, ^***^P < 0.001. All results are presented as mean ± standard deviation.

## 3 Results

### 3.1 DOP alleviates HFD-induced obesity, dyslipidemia, and hepatic steatosis

**Figure 1:**
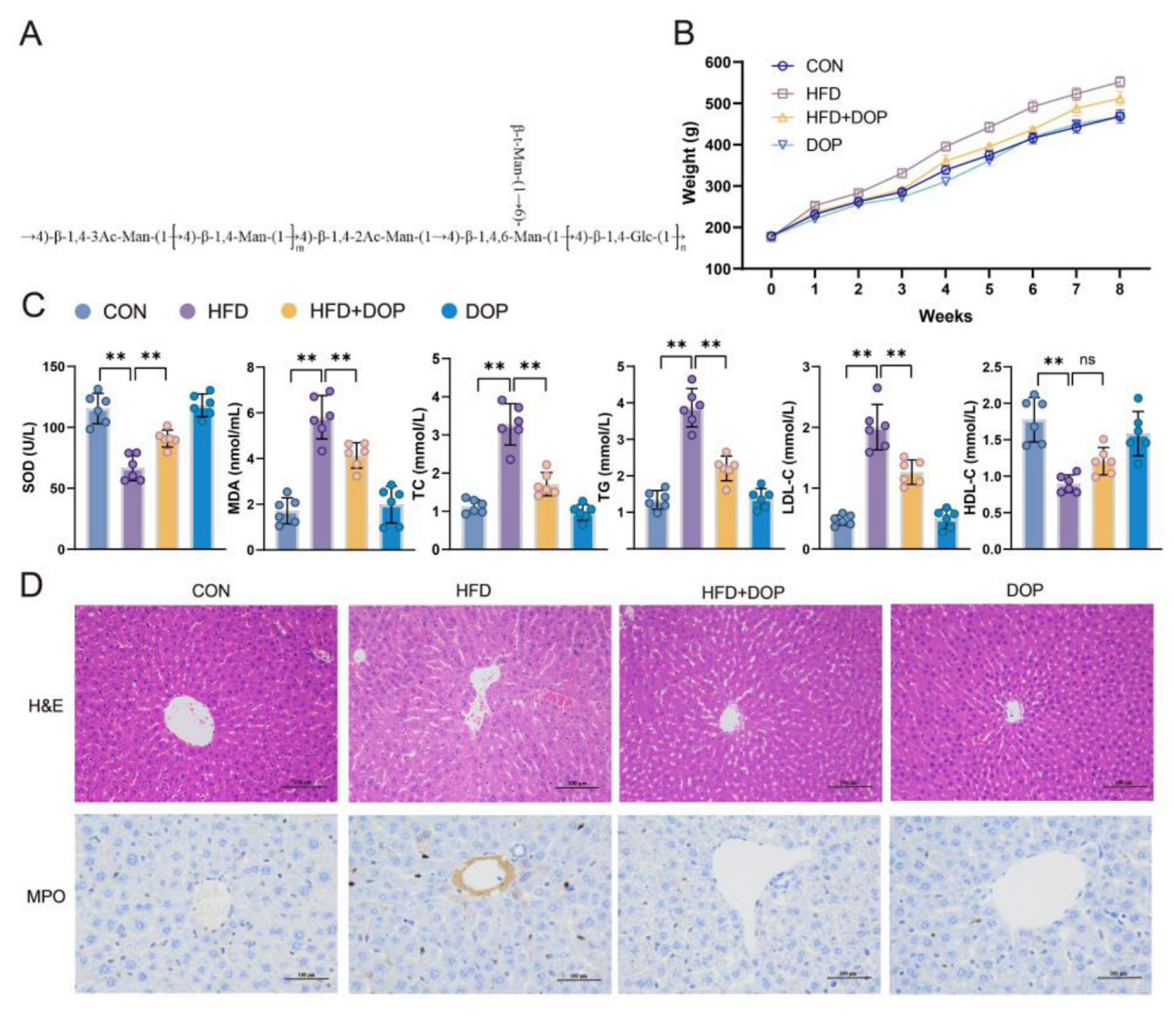
Effects of DOP on body weight and biochemical indices in HFD-fed rats. **(A)** Polysaccharide structure representation. **(B)** Body weight changes throughout the study period. **(C)** Serum concentrations of SOD, MDA, TC, TG, LDL-C, and HDL-C measured using commercial kits (**P < 0.01; ns, not significant; *n* = 3). **(D)** Representative images of liver sections stained with H&E and immunohistochemical staining for MPO.

Consistent with the obesogenic effect of the high-fat diet (HFD), we observed significant body-weight gain in the HFD group, which was markedly attenuated by DOP treatment (Figure 1A). DOP also mitigated HFD-induced dyslipidemia, as evidenced by lower serum TC, TG, and LDL-C, together with elevated HDL-C (Figure 1B). Moreover, DOP reversed the HFD-associated oxidative stress, reflected by restored SOD activity and reduced MDA levels (Figure 1B). Histopathology by H&E staining showed normal hepatocyte morphology in the CON group, whereas the HFD group displayed marked hepatocellular ballooning, edema; these pathological features were substantially alleviated by DOP (Figure 1C). Immunohistochemistry further demonstrated that DOP partially reduced the expression of MPO in the liver of the HFD group (Figure 1D).

### 3.2 LC–MS analysis detects elevated metabolites in plasma of HFD-DOP

**Figure 2:**
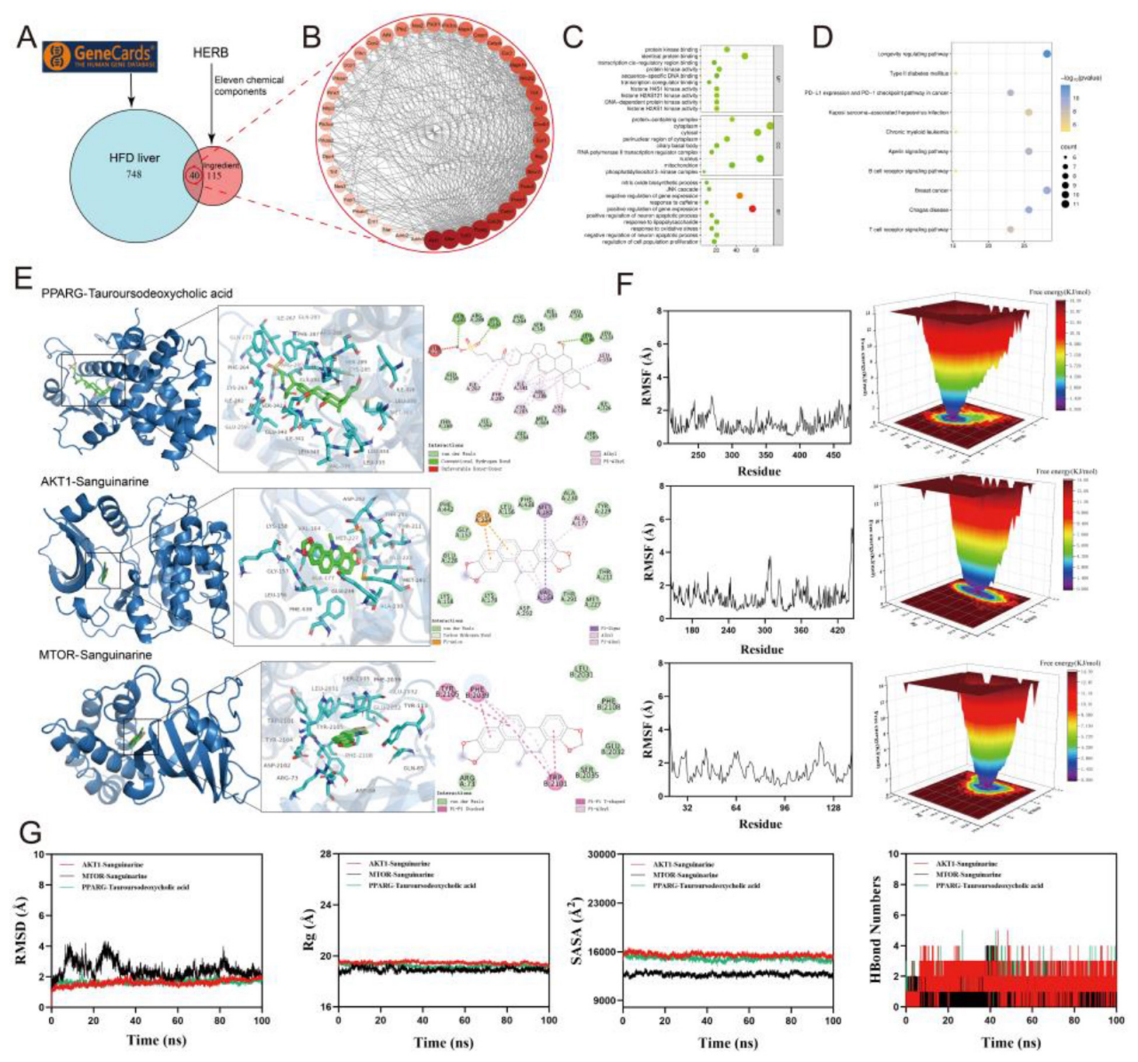
Network pharmacology analysis. **(A)** Venn diagram of overlapping targets between HFD liver and DOP plasma metabolites; **(B)** protein–protein interaction (PPI) network; **(C)** GO enrichment analysis; **(D)** KEGG pathway analysis; **(E)** molecular docking; **(F)** The RMSF values and free energy landscape diagram of the protein-ligand complex. **(G)** Molecular dynamics simulation of protein-ligand complexes.

LC-MS analysis detected a total of 186 metabolites that had a higher level in the plasma of HFD-DOP group in comparison with HFD group, among which two key metabolites—tauroursodeoxycholic acid and sanguinarine—showed significant elevation (Supplementary Figure S3). These results suggest that DOP may modulate specific metabolic pathways, thereby influencing the overall metabolic profile of the rats. Potential targets were identified by retrieving data from the HERB and GeneCards databases to determine overlapping targets. Subsequently, Cytoscape was employed to construct the network and screen for hub genes based on degree centrality, including PPARG, AKT1, and MTOR (Figures 2A, B). The enrichment analysis revealed that the targets are significantly enriched in pathways such as the B cell receptor signaling pathway and the T cell receptor signaling pathway, while the GO analysis highlighted biological processes including response to oxidative stress and the JNK cascade (Figures 2C, D). Molecular docking analysis revealed that DOP can bind strongly to key liver targets involved in metabolism and stress response, including PPARG with a binding energy of −8.7 kcal/mol, AKT1 with −9.3 kcal/mol, and MTOR with −12.0 kcal/mol. These targets play crucial roles in lipid metabolism, cell proliferation, and stress regulation, suggesting that DOP may exert its effects by modulating these core proteins. Furthermore, molecular dynamics simulations demonstrated the stability of the protein-ligand complexes, showing sustained hydrogen bonding and conformational stability over 100 ns. This supports the hypothesis that DOP can bind stably to these key proteins, potentially regulating pathways related to lipid metabolism and stress response in the liver. In summary, DOP may alleviate high-fat diet-induced liver damage by interacting with PPARG, AKT1, and MTOR, thereby modulating hepatic lipid metabolism and stress response pathways (Figures 2E–G).

### 3.3 DOP intervention alters the metabolic activity in the liver of HFD

**Figure 3:**
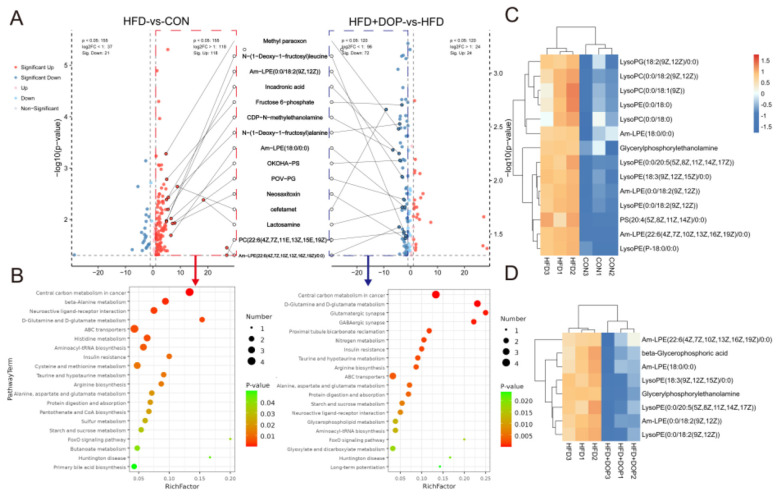
Liver metabolomics. **(A)** Volcano plots of the HFD vs. CON and HFD+DOP vs. HFD comparison groups (*P* < 0.05, VIP > 1); **(B)** TOP20 KEGG-enriched bubble plots for the upregulated metabolites in the HFD vs. CON comparison and the downregulated metabolites in the HFD+DOP vs. HFD comparison; **(C)** Significantly altered Glycerophosphocholines in the HFD vs. CON comparison; **(D)** Significantly altered Glycerophosphocholines in the HFD+DOP vs. HFD comparison.

Further investigation focused on analyzing metabolic alterations in liver tissues, aiming to clarify the specific role of DOP in hepatic metabolic regulation to understand its modulating effects on lipid metabolism. LC-MS analysis revealed distinguished patterns between the CON, HFD, and HFD+DOP groups acquired in positive and negative ionization modes, indicating a metabolic profile alteration induced by HFD and DOP intervention. Compared to the CON group, 155 differential metabolites (VIP > 1, *P* < 0.05) were detected in the HFD group in comparison with CON group. DOP intervention resulted in 120 differential metabolites compared to the HFD group (Figure 3A). KEGG pathway enrichment analysis highlighted significant alterations in the pathway of glycerophospholipid metabolism among other pathways (Figure 3B). Strikingly, 14 glycerophosphocholine metabolites were upregulated in the HFD group compared to controls; while DOP treatment reversed the upregulation of 7 of the 14 metabolites (Figures 3C, D), suggesting an effect on phospholipid metabolism, and these are Am-LPE(0:0/18:2(9Z,12Z)), Am-LPE(18:0/0:0), Am-LPE(22:6(4Z,7Z,10Z,13Z,16Z,19Z)/0:0), LysoPE(0:0/18:2(9Z,12Z)), LysoPE(0:0/20:5(5Z,8Z,11Z,14Z,17Z)), LysoPE(18:3(9Z,12Z,15Z)/0:0), Glycerylphosphorylethanolamine.

### 3.4 Transcriptome analysis reveals the effects of DOP on inflammatory and metabolic pathways

**Figure 4:**
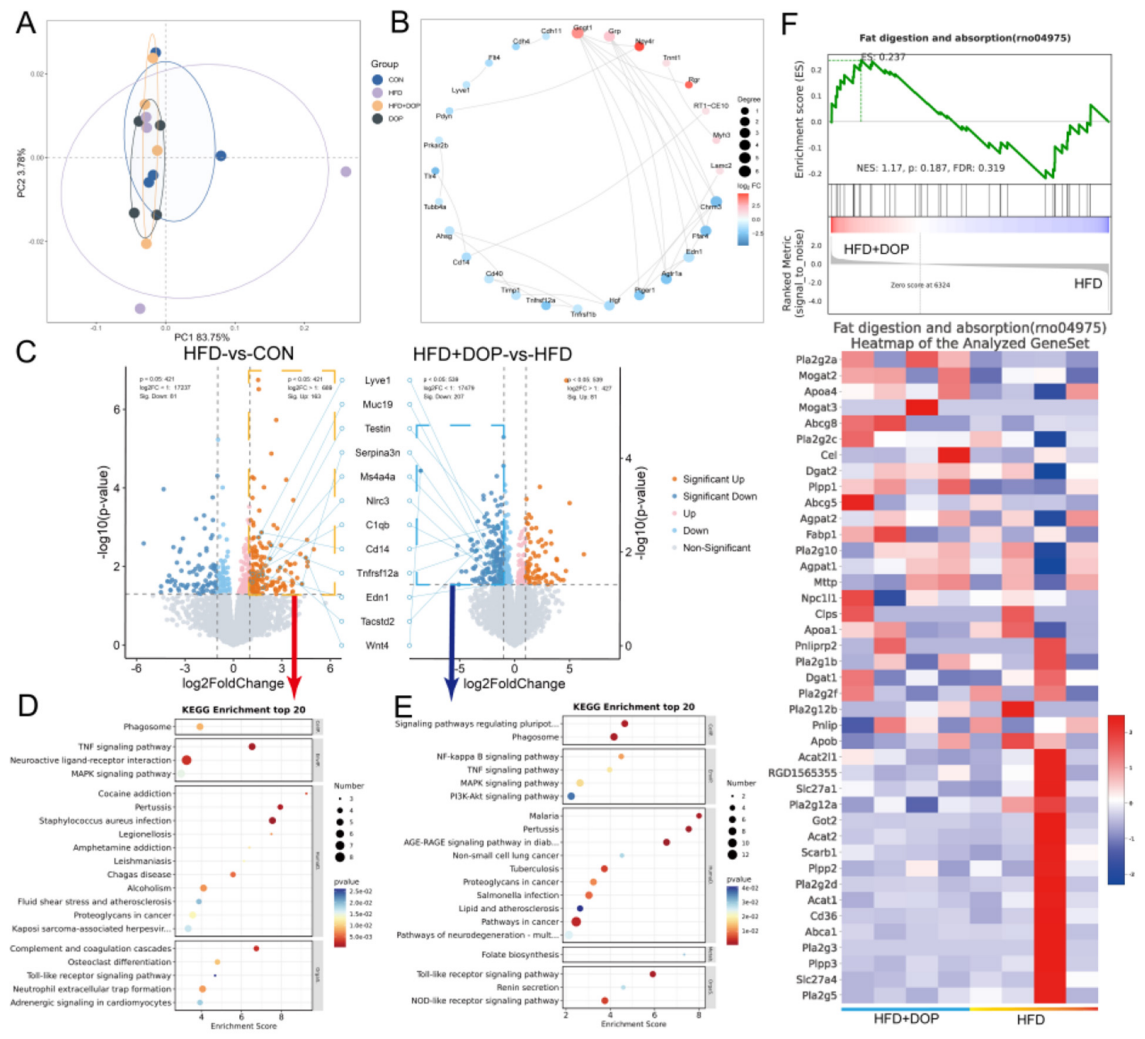
Liver transcriptome analysis. **(A)** Principal coordinate analysis (PCOA); **(B)** Protein-protein interaction (PPI) network of the top 30 genes; **(C)** Double volcano plot; **(D, E)** KEGG pathway enrichment analysis; **(F)** Gene set enrichment analysis (GSEA).

Despite the hepatic lipid metabolic changes described above, the principal coordinates analysis of liver transcriptomes failed to detect a clear difference among the groups, suggesting that DOP's effects may be mediated through a specific pathway rather than a global transcriptional change (Figure 4A). A protein-protein interaction (PPI) network constructed from the top 30 differentially expressed genes highlighted key hubs, including TLR4, CD14, CD40, TNFRSF12A, and TNFRSF1B which were downregulated after DOP intervention (Figure 4B). Volcano plots visualized transcripts with the largest differences between the control and HFD groups, as well as between the HFD and HFD+DOP groups (Figure 4C). KEGG enrichment analysis of differentially expressed genes revealed significant downregulation of inflammation-related pathways, including TNF, NF-κB, and MAPK signaling, following DOP treatment (Figures 4D, E). Gene Set Enrichment Analysis (GSEA) demonstrated enrichment of the genes associated with fat absorption in the HFD group treated with DOP, suggesting DOP may ameliorate hepatic lipid metabolism disorders through regulating these pathways (Figure 4F).

### 3.5 DOP modulates gut microbiota homeostasis and gut–liver crosstalk under a HFD

**Figure 5:**
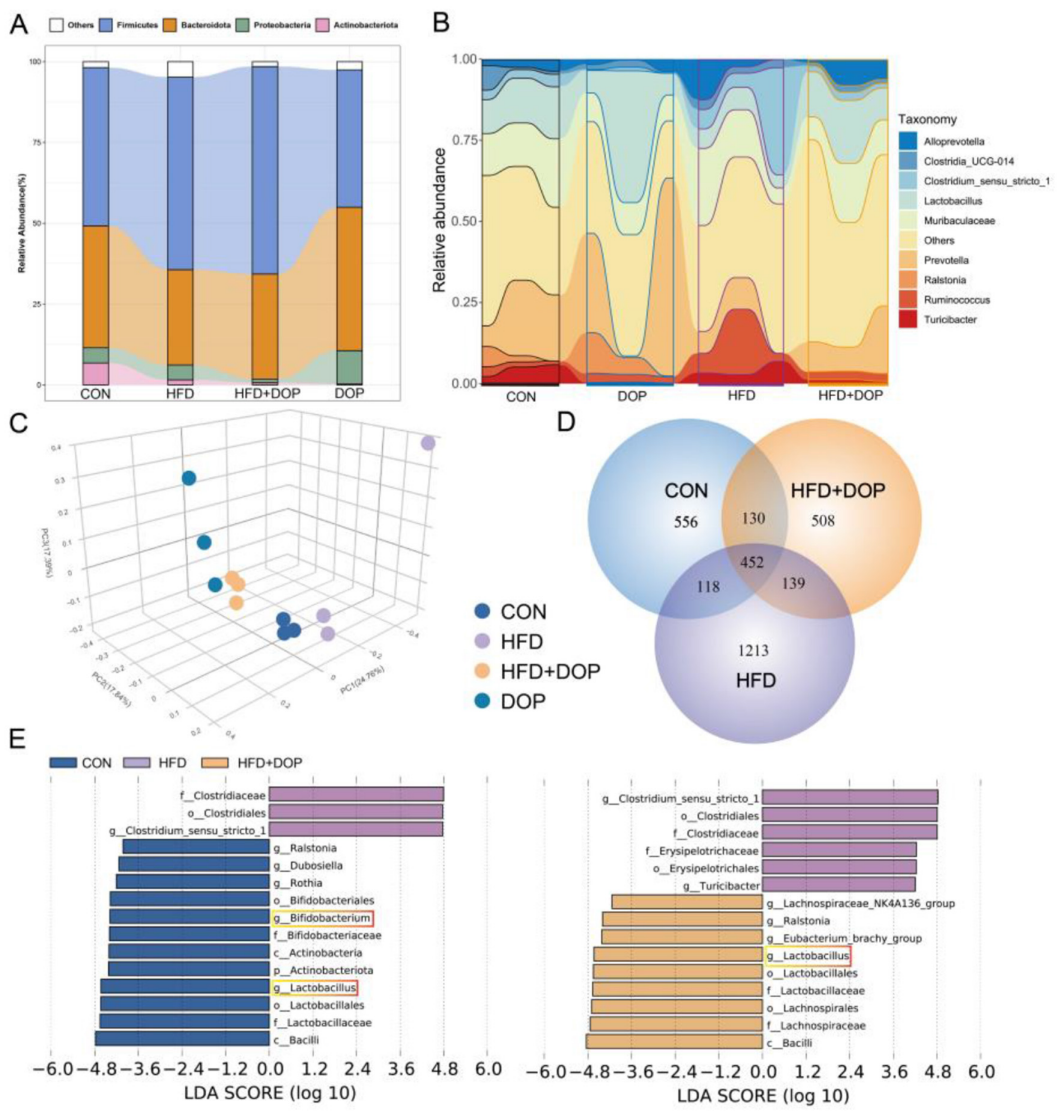
DOP regulates the composition of the intestinal microbiota in HFD rats. **(A, B)** Relative abundance of bacterial communities at the phylum level **(A)** and genus level **(B)** in different groups. **(C)** PCoA diagram based on Bray-Curtis distance. **(D)** Venn diagram. **(E)** LEfSe.

To assess whether DOP treatment affected the homeostasis of the gut microbiome, the samples collected from gut were subjected to 16S rRNA sequencing. Bar plots illustrated compositional shifts at both the phylum and genus levels upon DOP treatment (Figures 5A, B). Principal coordinates analysis (PCoA) shows that there are obvious distinctions among the groups (Figure 5C). Venn diagrams detailed the number and overlap of operational taxonomic units (ASVs) (Figure 5D). Linear discriminant analysis Effect Size (LEfSe) identified specific taxa altered in the HFD model, which is affected by DOP intervention. Notably, HFD reduced the abundance of beneficial bacteria such as *Bifidobacterium* and *Lactobacillus*, while increased *Clostridium sensu stricto 1*. Whereas DOP intervention significantly restored the HFD resulted loss of *Lactobacillus* (Figure 5E), indicating a prebiotic-like effect.

**Figure 6:**
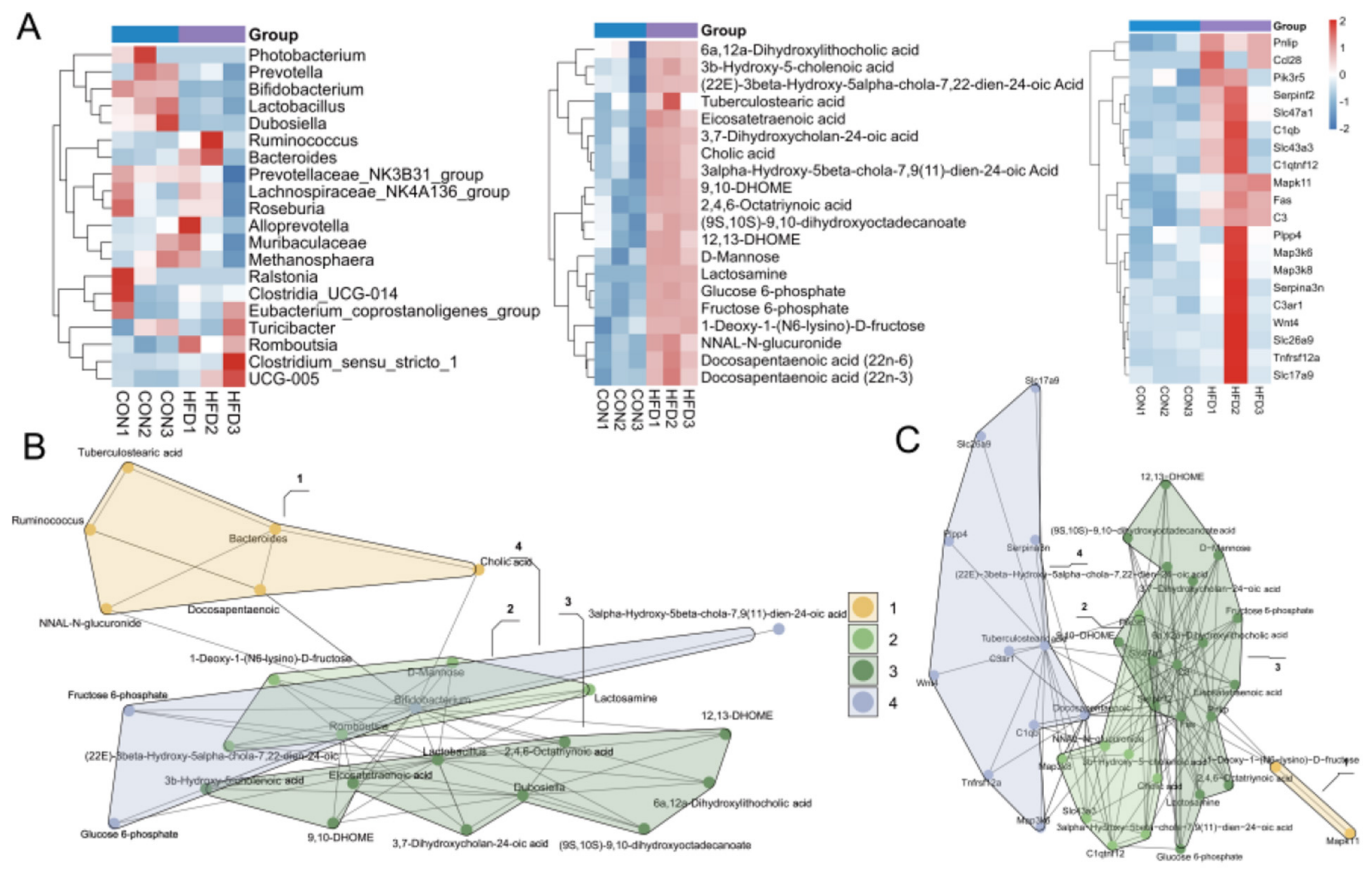
Key 20 microbiota, metabolites, and genes in the HFD vs. CON comparison and their correlation network. **(A)** Heatmap showing the key 20 microbiota, metabolites, and genes. **(B, C)** The key 20 microbiota, metabolites, and genes identified between the HFD and CON groups, and their corresponding correlation network.

**Figure 7:**
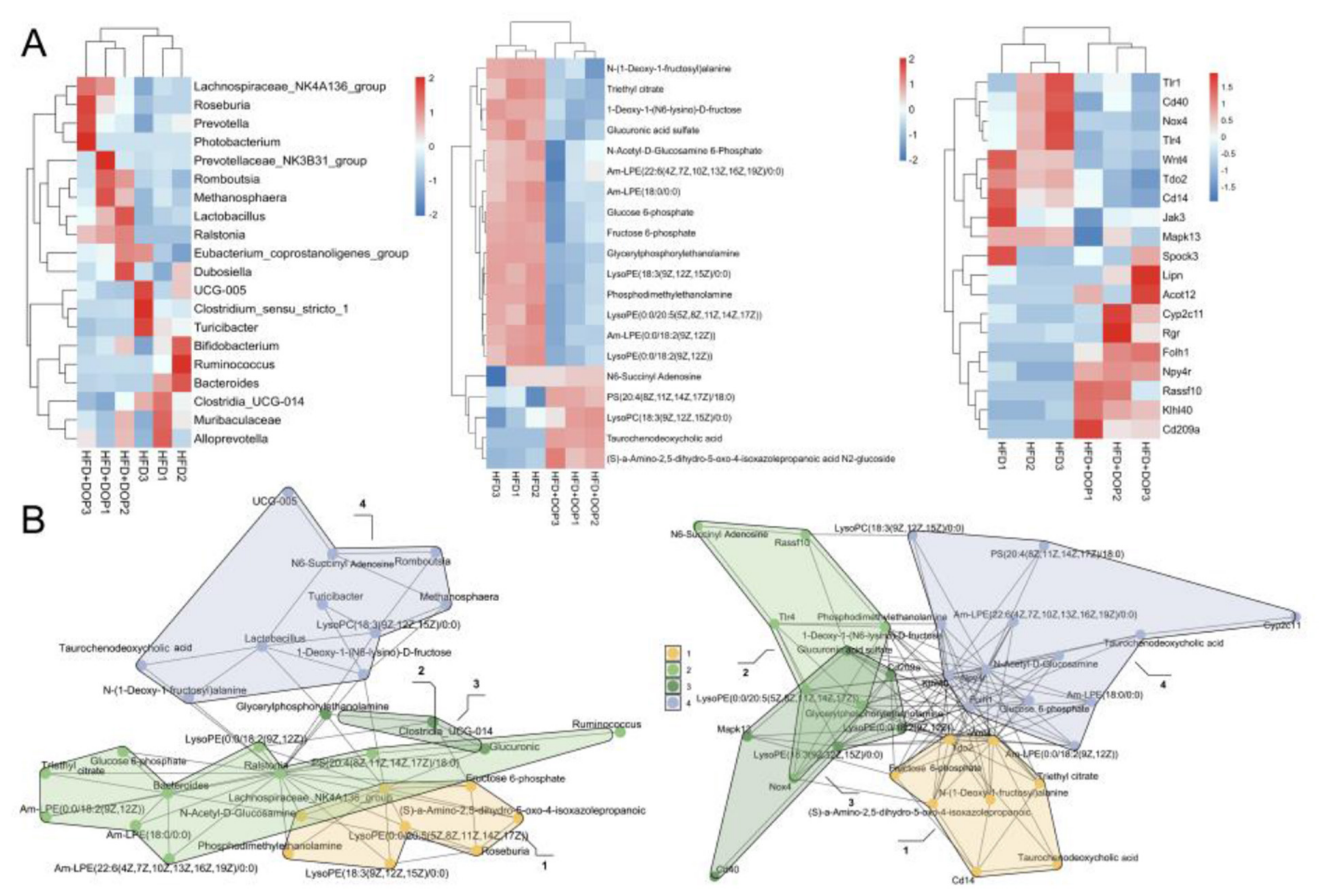
Key 20 microbiota, metabolites, and genes in the HFD+DOP vs. HFD comparison and their correlation network. **(A)** Heatmap showing the key 20 microbiota, metabolites, and genes. **(B, C)** The key 20 microbiota, metabolites, and genes identified between the HFD+DOP and HFD groups, and their corresponding correlation network.

Integrated analysis of gut microbiota, hepatic metabolome, and transcriptome data identified the top 20 key features that discriminate among the comparisons of HFD vs. CON (Figure 6A) and HFD+DOP vs. HFD (Figure 7A). A correlation-network analysis visualized the complex interactions among the measured features. In the HFD vs. CON comparison, the network revealed strong associations between *Lactobacillus* and glycolytic intermediates such as glucose-6-phosphate and fructose-6-phosphate. These metabolites also showed robust co-correlations (r > 0.8) with C3 and with Slc47a1, FAS, Pnlip, and Serpinf2, and their levels were upregulated under HFD. In the HFD+DOP vs. HFD comparison, *Lactobacillus* exhibited strong associations with N6-Succinyl Adenosine, 1-Deoxy-1-(N6-lysino)-D-fructose, LysoPC(18:3(9Z,12Z,15Z)/0:0), Glycerylphosphorylethanolamine, Taurochenodesoxycholic acid, and N-(1-Deoxy-1-fructosyl) alanine. Moreover, taurochenodeoxycholic acid, glucose-6-phosphate, fructose-6-phosphate, Npy4r, Tdo2, and Wnt4 showed significant co-associations (r > 0.8). Collectively, these patterns imply that DOP-modulated gut–liver crosstalk contributes to mitigating HFD-induced metabolic dysregulation and inflammation by orchestrating host–microbiome interactions along the gut–liver axis (Figures 6, 7).

### 3.6 DOP activates the SIRT6/PGC-1α pathway to mitigate oxidative stress and lipid accumulation

**Figure 8:**
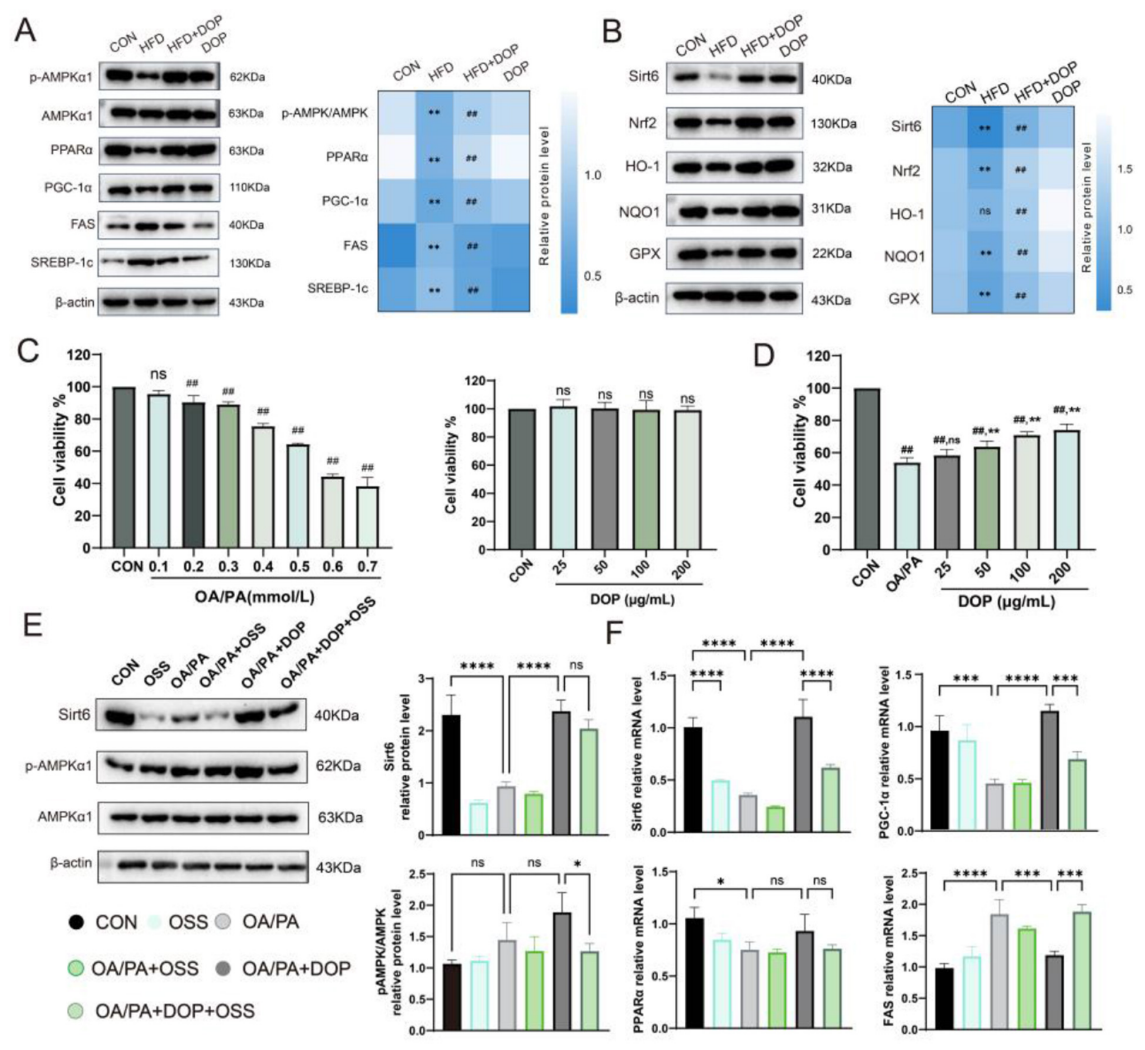
DOP alleviates hepatic lipid disorder and oxidative damage via the SIRT6/PGC-1α pathway. **(A, B)** Protein expression levels of SIRT6, NRF2, HO-1, NQO1, GPX, p-AMPK, AMPK, PPARα, PGC-1α, FAS, and SREBP-1c were determined by Western blotting. **P < 0.01: HFD vs. CON group; ^##^*P* < 0.01: HFD+DOP vs. HFD group (*n* = 3). **(C, D)** Cell viability was assessed by CCK-8 assay. **(E)** Protein expression of SIRT6, p-AMPK, and AMPK was detected by Western blotting. **(F)** mRNA expression levels of SIRT6, PGC-1α, FAS, and PPARα were measured by RT-qPCR. *P < 0.05, ***P < 0.001, and ****P < 0.0001.

To dissect the molecular mechanism, we examined key components of the SIRT6-centered signaling axis. Western blot analysis revealed that DOP increased the protein levels of SIRT6, NRF2 and its downstream targets HO-1 and NQO1, as well as GPX, phosphorylated AMPK (p-AMPK), PPARα, and PGC-1α; concomitantly, DOP decreased the lipogenic enzyme FAS (Figures 8A, B, E, F). To validate the hypothesis that DOP attenuates oxidative stress and lipid dysregulation via activating the SIRT6/PGC-1α/PPARα lipid-oxidation axis, we used an OA/PA-induced hepatocyte injury model (IC50 ≈ 0.6 mM). In this model, DOP significantly rescued OA/PA-induced decreases in cell viability in a concentration-dependent manner (Figures 8C, D). Importantly, DOP counteracted the OA/PA-induced reduction in SIRT6 levels, and co-treatment with the SIRT6-specific inhibitor OSS-128167 attenuated the protective effect of DOP and the upregulation of PGC-1α (Figures 8E, F), indicating that the protective actions of DOP are SIRT6-dependent with PGC-1α acting downstream. Collectively, these findings support a model in which DOP mitigates oxidative stress and lipid accumulation through SIRT6-dependent activation of NRF2 and SIRT6/PGC-1α pathways.

## 4 Discussion

This study systematically demonstrated the significant therapeutic effect of DOP in improving NAFLD induced by HFD, and elucidated that it functions through two key mechanisms: direct activation of the liver SIRT6/PGC-1α signaling axis and indirect protection through remodeling of the intestinal microbiota structure. The pharmacological activity of DOP not only corrected the imbalance of liver lipid homeostasis, but also improved metabolic dysfunction by stabilizing the microbial community, which is consistent with the research results of polysaccharide-mediated metabolic improvement (40, 41). This study further provided empirical evidence indicating that DOP can effectively restore the colonization of *Lactobacillus* in the intestine (42), and the enrichment of *Lactobacillus* has been proven to be helpful in restoring intestinal metabolites and alleviating fatty liver diseases associated with metabolic dysfunction (43).

One of the core pathological features of non-alcoholic fatty liver disease (NAFLD) is the abnormal accumulation of lipids within liver cells. This process involves the coordinated dysregulation of various lipid metabolisms, including glycerophospholipids (44). Hepatic metabolomic profiling shows that HFD exposure significantly upregulates glucose-6-phosphate, fructose-6-phosphate, and specific glycerophosphocholine subspecies. Extensive studies have established a causal link between dysregulated glycerophospholipid metabolism and prolonged HFD patterns (45, 46). The metabolism of glycerophospholipids generates bioactive molecules, which not only cause endothelial dysfunction, lipid accumulation and myocardial cell damage, but also regulate inflammatory and oxidative stress responses (47). Furthermore, elevated levels of glycolytic intermediates reflect both increased hepatic glucose utilization and impaired carbohydrate homeostasis, underscoring the central role of dysregulated energy metabolism in the development and progression of hepatic steatosis.

Following DOP administration, metabolomic profiling revealed a significant attenuation of glucose-6-phosphate, fructose-6-phosphate, and specific glycerophosphocholines, e.g., notably LysoPE(0:0/18:2(9Z,12Z)), LysoPE(0:0/20:5(5Z,8Z,11Z,14Z,17Z)), LysoPE(18:3(9Z,12Z,15Z)/0:0), effectively reversing the HFD-induced dysregulation. In contrast, levels of tauroursodeoxycholic acid (TUDCA) were markedly increased. TUDCA, an FDA-approved therapeutic for chronic cholestasis, is well-documented for its dual hepatoprotective and metabolic regulatory properties in diabetes and obesity (48–50). By integrating metabolomic screening with molecular docking simulations, we identified TUDCA as a potential key endogenous mediator of DOP's effects, with *in silico* modeling demonstrating its high-affinity binding to the ligand-binding domain of PPARγ. Collectively, these data suggest that DOP exerts its hepatoprotective effects by reprogramming lipid metabolism and modulating the enterohepatic circulation of bile acids, specifically through the upregulation of TUDCA.

To delineate the key pathways by which DOP regulates glucolipid metabolism, our study demonstrates that DOP intervention orchestrates a multifaceted hepatic response. It transcriptionally represses inflammatory mediators (TLR4, NOX4) and the pattern recognition receptor CD14, and the co-stimulatory molecule CD40, thereby alleviating inflammation and oxidative stress. Concurrently, it corrects lipid metabolic disturbances by upregulating PPARα/PGC-1α transcriptional complexes and suppressing master lipogenic regulators (FAS, SREBP-1c). To further explore its upstream regulatory mechanism, we discovered that SIRT6 is the key molecular target mediating the aforementioned multiple protective effects of DOP. SIRT6 can activate the NRF2 signaling pathway, drive its nuclear translocation, and initiate the cascade transcription of downstream antioxidant enzyme genes, including HO-1 and NQO1, thereby constructing a powerful antioxidant defense system (51). Previous studies have shown that DOP enhances hepatic antioxidant defenses via the NRF2/HO-1 cascade, collectively mitigating lipid accumulation (32, 52, 53). Pharmacological activation of SIRT6 by DOP orchestrates a synergistic induction of PGC-1α and NRF2, driving mitochondrial β-oxidation capacity and oxidative phosphorylation efficiency alongside enhanced glutathione cycling, which collectively counteracts HFD-induced steatotic progression (54). Treatment of hepatocytes with OSS_128167, a SIRT6 inhibitor abrogated DOP's beneficial effects on lipidomic homeostasis and oxidative damage repair, provided definitive evidence of the SIRT6/PGC-1α axis as the mechanistic linchpin in DOP's hepatoprotective paradigm (55). It is worth noting that recent studies have revealed the crucial role of the SIRT1-SMPD3 axis in the progression of MASH - DNA damage upregulates SMPD3 expression by inhibiting SIRT1 activity, promoting ceramide accumulation and liver damage (56). SIRT6 and SIRT1 belong to the same third class of deacetylase family and have complementary functions in metabolic regulation. Indirectly regulating sphingolipid metabolism through activating SIRT6 may be a potential regulatory pathway for DOP to exert its protective effect on liver injury.

Integrated correlation analysis demonstrated that dynamic shifts in *Lactobacillus spp*. colonization status exhibited strong covariation with critical nodes of the enterohepatic metabolome, particularly taurochenodeoxycholic acid flux and glycolytic intermediates (glucose-6-phosphate/fructose-6-phosphate). These data implicate gut microbial ecology as a potential orchestrator of DOP's hepatoprotective efficacy via metabolic crosstalk regulation. Although our work mechanistically delineates the SIRT6-dependent signaling circuitry activated by DOP in hepatocytes, the causal cascade initiating from microbial community restructuring to epigenetic reprogramming in hepatic parenchyma remains enigmatic. Future studies involving fecal microbiota transplantation (FMT) in germ-free mice or microbial metabolite reconstruction will be crucial for subsequent mechanism analysis, in order to reveal the dynamic changes in the spatial and temporal aspects of the regulatory role of DOP in the pathogenesis of non-alcoholic fatty liver disease (NAFLD), and thereby clarify the underlying metabolic network of its therapeutic effect.

## 5 Conclusion

In summary, this study has established DOP as a potential natural product for the intervention of NAFLD induced by HFD. Its mechanism of action involves the regulation connecting the intestinal microbiota and metabolism. Firstly, DOP reshapes the intestinal microecology by enriching beneficial bacteria such as *Lactobacillus* and regulating the intestinal-liver metabolic profile (such as upregulating TUDCA). Secondly, we identified and verified that SIRT6 is a potential molecular switch in the liver; DOP activates the SIRT6/PGC-1α signaling axis, inhibits lipid production and enhances NRF2-mediated antioxidant defense. The SIRT6 inhibitor OSS_128167 eliminates these beneficial effects, precisely confirming the SIRT6-dependent action of DOP. In conclusion, these findings elucidate a complex network involving metabolic changes, gene regulation and microbial regulation, providing a solid theoretical basis for developing DOP as a targeted nutritional or pharmacological intervention for metabolic liver diseases.

## Data Availability

The 16S rRNA sequencing data and transcriptomics data presented in the study are available in the NCBI under accession numbers PRJNA1469440 and PRJNA1471211 respectively. The Metabolomics data presented in this study are available in MetaboLights under accession number MTBLS14575.
