# Supplementary material for: Dendrobium officinale polysaccharide ameliorates high-fat diet-induced hepatic lipid metabolic disorder via the SIRT6/PGC-1α signaling axis
Source: Front Nutr. 2026 Jun 2;13:1763341. doi: 10.3389/fnut.2026.1763341 (PMC13271076; doi:10.3389/fnut.2026.1763341)
Supplement: Supplementary file 1 [file Supplementary_file_1.docx]

**1. Extraction and purification of DOP**

Dried *Dendrobium officinale* (500 g) was subjected to hot water extraction. A volume of water in ten times of the plant material was then added (v/w, folloed by a boiling water extraction that lasted for three hours. This step was repeated two more times. Impurities were removed from the resulting supernatant through filtration, and the solutes were then concentrated by precipitation with four times the volume of anhydrous ethanol (with thorough stirring). To facilitate precipitation, the mixture was refrigerated at 4°C overnight. The precipitate was collected using a Brinell funnel and filter paper, and then dissolved in distilled water. To facilitate dissolution and remove residual ethanol, the sample was incubated in a water bath maintained at 60°C. Finally, the dissolved sample was freeze dried to yield the final product. As described above, our isolation process for the crude polysaccharide included separation and purification via both aqueous extraction and alcohol precipitation. This process was designed and implemented to ensure uniformity in terms of molecular weight and polarity.

After isolation, polysaccharide was purified on a DEAE-52 anion-exchange column. DEAE-52 fillers were first immersed in hydrochloric acid (0.5 mol/L) for 1 h an then washed with 4–5 volumes of distilled water to remove any impurities. These processes were repeated several times until neutralization was achieved. The DEAE-52 anion-exchange column was then equilibrated with distilled water for two hours at a flow rate of 5 mL/min. Prior to loading, the crude polysaccharide was dissolved in distilled water, heated, and then centrifuged at 12000 rpm. The resulting supernatant was used as the sample for loading. This was performed with distilled water at a flow rate of 15 mL/min. After loading, four solvent elution runs were conducted: First, with three volumes of water; Second, with 0.2 M NaCl; Third, with 0.5 M NaCl; and fourth, with 1.0 M NaCl. Carbohydrate molecules in the eluate fractions were detected spectrophotometrically at 490 nm using the phenol-sulfuric acid method. Peak shape was taken into account for collection and concentration purposes. Finally, fractions of interest were transferred to a dialysis bag (molecular weight cutoff, 3500 Da) and extensively dialyzed against it. The recovered fractions were then freeze-dried for storage.

**2.Determination of molecular weight and monosaccharide composition of DOP**

The average molecular weight (Mn) and Mw values of DOP were 148079 Da and 150832 Da, respectively, giving a dispersion index (Mw/Mn) of 1.01859. Additionally, a pre-column derivatization of DOP hydrolysates was conducted using PMP, and the monosaccharide composition of DOP was then analyzed via HPLC. The results revealed that DOP was composed of mannose (Man) and glucose (Glc) residues (only) in a molar ratio of 79:21, confirming its classification as glucomannan. Polysaccharide components were further analyzed using infrared spectroscopy (Fig. S1B). An absorption band in the 3600–3200 cm^-1^ region corresponds to the stretching vibration absorption of -OH, a characteristic feature of sugars. The absorption peak at 3372 cm^-1^ represents the stretching vibration absorption peak of O-H, also characteristic of sugar. Additionally, the peaks observed at 2921 cm^-1^ and 2884 cm^-1^ correspond to the stretching vibration absorption peaks of C-H, which are again distinctive features of sugars. The prominent absorption peaks at 1731 cm^-1^ and 1641 cm^-1^ can be attributed to C=O stretching vibrations. The distinct absorption peaks at 1423 cm^-1^, 1151 cm^-1^, and 1064 cm^-1^ can be ascribed to C-O stretching vibrations. Another absorption peak at 1373 cm^-1^ can be attributed to C=O symmetric stretching vibrations. The absorption peaks at 1309 cm^-1^, 1245 cm^-1^, and 1027 cm^-1^ can be attributed to O-H variable angle vibrations. An absorption peak at 950 cm^-1^ could be attributed to the roll vibration of the terminal methylate of the pyran ring. Another absorption peak at 902 cm^-1^ could be attributed to the stretching vibration of the asymmetric ring of the pyrane ring. An absorption peak at 873 cm^-1^ could be attributed to the C-H angular oscillation of the end group of the pyrane ring to the equatorial bond other than isomeric C-H. Finally, an absorption peak at 808 cm^-1^could be attributed to pyran ring symmetric ring stretching vibrations.


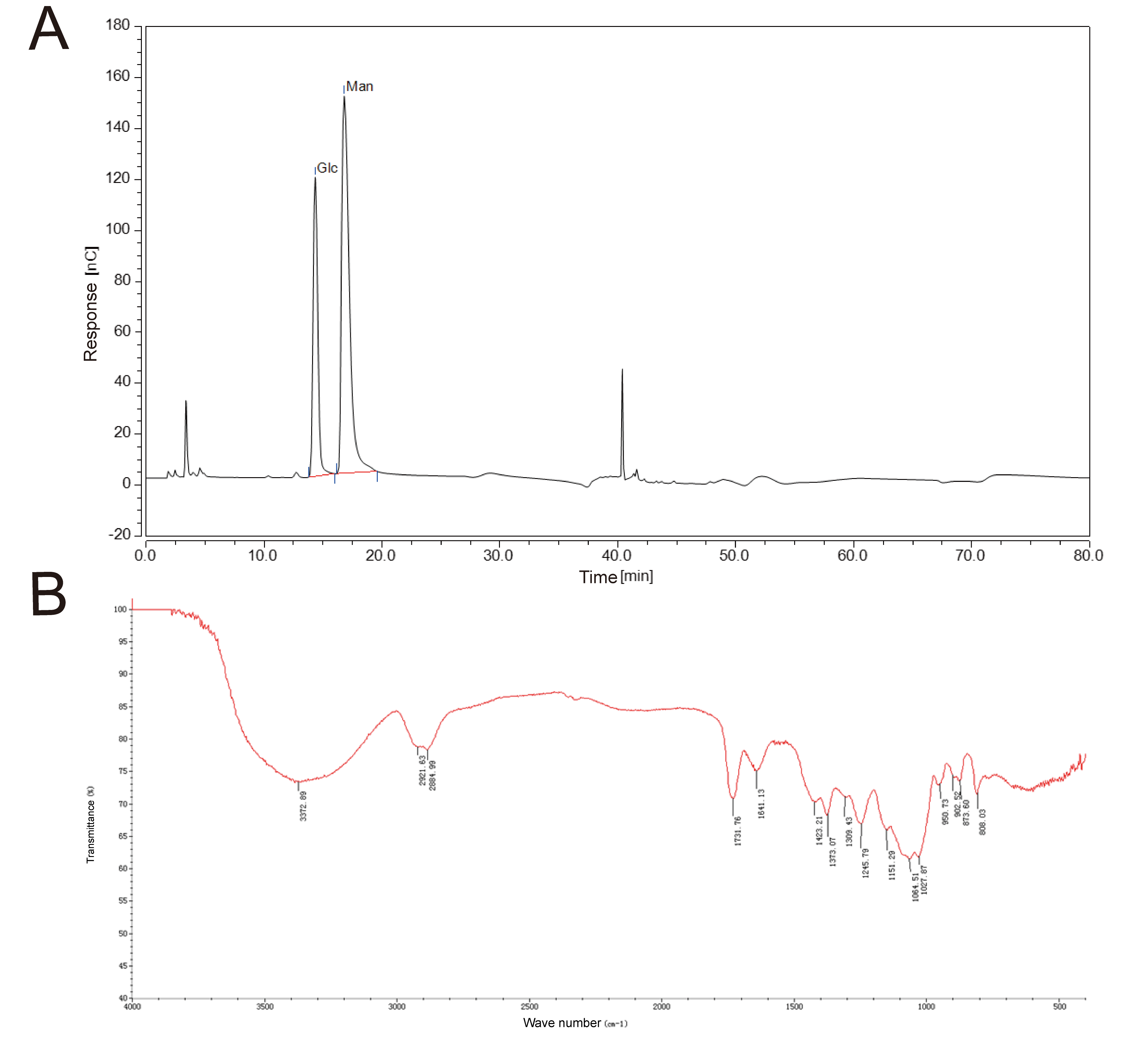


**Figure S1. Determination of molecular weight and monosaccharide composition of DOP, Molecular weight and purity of polysaccharides were determined by HPGPC.** (A) High-performance liquid chromatography (HPLC) chromatogram of 1-phenyl-3methyl-5-pyrazolone (PMP)-derived DOP; (B) Infrared spectrum.

**3. Methylation and Nuclear magnetic resonance analysis of DOP**

Methylation tests revealed that the two constituent monosaccharides comprising DOP exhibit multiple residue forms. Among these, 1,4-Man was found to be the predominant form and can be considered the principal component of the main chain. The other monosaccharide was glucose, which forms residues such as 1,4-Glc and t-Glc. These two residues are presumed to contribute to both the main and branched chains. Based on our composition, proportion, and molecular weight analyses of methylated sugar residues, it is evident that DOP possesses a prominent main chain structure with a relatively low presence of branched chains (Table 1).

The structure of DOP was confirmed through the analysis of multiple NMR one-dimensional and two-dimensional spectra. The 1H NMR and ^13^C NMR spectra of DOP are presented in Figure 2. In the ^1^H NMR spectrum of DOP (Fig. S2A), considering the higher abundance of mannose, the allocephalic hydrogen may overlap with heavy water at the 4.71 ppm position. To address this, allocephalic information was determined by combining two-dimensional spectra. Within the 3.0 – 4.3 ppm range in the hydrogen spectrum, alkyl proton signals outside the heterohead hydrogen were eliminated from residual sugar structures resulting in a more diverse signal pattern within this region, which is a characteristic feature observed in polysaccharide hydrogen spectra. Four prominent heterologous proton signals were identified at δ5.43, 5.15, 4.57, and 4.45 ppm in the low field region. The signal observed at δ2.1 ppm in the high field region is attributed to the methyl proton of the acetyl group within the polysaccharide structure, indicating a higher degree of acetylation and the presence of various forms. The signal at δ4.70 ppm corresponds to solvent heavy water, while the methyl proton signal at δ1.84 ppm represents the internal standard acetone. In the ^13^C NMR spectrum of DOP (Fig. S2B), a chemical shift near δ173 ppm suggests the presence of -o-acetyl, whereas a signal near δ22 ppm can be assigned to the methyl carbon of the acetyl group. Notably, four significant anomalous carbon signals were detected in the low field region of the carbon spectrum at δ102.49, 100.51, 95.89, and 92.07 ppm.

In the HSQC spectrum of DOP (Fig. S2C), six prominent overlapping peaks were observed at δ4.85/99.11, 4.78/99.54, 4.71/99.16, 4.67/99.96, 4.41/102.89, and 4.44/102.49 ppm in the heterojunction signal region, indicating that DOP is predominantly comprised of six distinct types of sugar residue (termed M, M2, M3, M46, Mt, G4). The position of H2 in sugar residue M was determined based on the cross peak at δ 4.67/4.03 ppm observed in the COSY spectrum (Fig. S2D). Additionally, C2 was confirmed by the cross-peak signal at δ 4.03/69.98 ppm detected in the HSQC spectrum. Analysis of COSY and HSQC spectra revealed cross peaks at δ3.73/71.48, 3.72/76.37, 3.38/74.71, and 3.65 (3.83)/60 .19 ppm, which were attributed to the positions of H3/C3-H6/C6 signals in sugar residue M. The structure of sugar residue M was determined as →4)-b-D-Manp-(1→ ^[31]^. Furthermore, using COSY, NOESY, and HSQC spectra analyses, chemical shifts for the H1/C1-H6/C6 positions of the other five sugar residues were also investigated. These results are summarized in Table 2.

Compared with sugar residue M, the H2 of sugar residue M2 exhibited a significant chemical shift to the lower field (5.43 ppm), indicating that the hydroxyl group is acetylated at this position. Therefore, sugar residue M2 was determined to be →4) -2Ac-b-D-Manp-(1→. From the 5.43/2.11 ppm cross peak in the NOESY spectrum, the signal of the acetyl matrix at the H2 position was determined to be 2.11 ppm, and the signal of acetyl carbonyl carbon was determined to be 172.95 ppm by HMBC. For sugar residue M3, the analysis was similar to that of sugar residue M2, with a signal of 5.03 ppm at H3, confirming the presence of acetylation substitution at the H3 position, giving a →4) -3Ac-b-D-Manp -(1→ mannose sugar residue, and confirming that the signal of the acetyl-matrix at the H3 position is 2.08 ppm. The signal of acetyl carbonyl carbon was determined to be 173.05 ppm by HMBC. Because the content of the Mt and M46 sugar residues is small, the location of the heterotopic was confirmed and the signal was attributed using previous research. The heterotopic signal of the G4 sugar residue was 4.44/102.49, and in its COSY diagram, the signal of H1/H2 appeared at 4.44/3.28, giving a →4) -b-D-Glcp -(1→ glucose sugar residue. It should be noted that the NMR HSQC spectra also revealed cross peaks of 5.15/92.07, 5.10/93.66, and 4.56/95.89 in the heterotopic region corresponding to monosaccharide heterotopic signals of free α-glucose, α-mannose, and β-glucose. Therefore, these results confirm the presence of a small amount of these three free monosaccharides in our isolated product.

The residual sugar linkage of DOP was analyzed using HMBC and NOESY spectra. In the HMBC spectrum (Fig. S2E), a correlation was observed between H1 (δ4.67 ppm) of sugar residue M and C4 (δ76.65 ppm) of sugar residue M or M2, indicating a potential connection. Additionally, the HMBC signal between H1 (δ4.78 ppm) of sugar residue M3 and C4 (δ76.37 ppm) of sugar residue M confirmed the presence of a linkage. The signaling between H1 (δ4.71 ppm) of sugar residue M46 and C4 (δ78.48 ppm) of sugar residue G4 provided evidence for the existence of a glycosidic bond between them. In the NOESY spectrum (Fig. S2F), the correlation signal at 4.67/3.73 confirmed the presence of a glucosidic bond between residue 1 and residue 4 in M's own structure, while the signal at 4.85/3.68 suggested the presence of a structural fragment corresponding to b-1, 4-2Ac-Man-(1→4)-b-1, 4, 6-Man-(1→. The signal at 4.74/3.57 predicts a b-t-Man-(1→6)-b-1, 4, 6-Man-(1→ fragment structure, and it confirms that the signal at 4.44/3.60 corresponds to the b-1, 4-Glc-(1→ sugar residue with four connections.

**Table 1. Methylation test results of glucose and glycan**

| RT | Methylated sugar | Mass fragments (m/z) | Molar ratio | Type of linkage |
| --- | --- | --- | --- | --- |
| 27.570 | 2,3,4,6-Me_4_-Glcp | 43,71,87,101,117,129,143,161,205 | 0.014 | Glcp-(1→ |
| 27.961 | 2,3,4,6-Me_4_-Manp | 43,71,87,101,117,129,143,161,205 | 0.050 | Manp-(1→ |
| 41.076 | 2,3,6-Me_3_-Manp | 43,87,99,101,113,117,129,131,161,173,233 | 0.675 | →4)-Manp-(1→ |
| 42.232 | 2,3,6-Me_3_-Glcp | 43,87,99,101,113,117,129,131,161,173,233 | 0.209 | →4)-Glcp-(1→ |
| 48.790 | 2,6-Me_2_-Manp | 43,87,99,117,129,149 | 0.010 | →3,4)-Manp-(1→ |
| 51.119 | 3,6-Me_2_-Manp | 43,87,99,113,129,173,189,233 | 0.013 | →2,4)-Manp-(1→ |
| 53.317 | 2,3-Me_2_-Manp | 43,71,85,87,99,101,117,127,159,161,201,261 | 0.029 | →4,6)-Manp-(1→ |

**Table 2. Attribution table presenting the major glucose and glycan residues**

| **Residues (Mark)** | **H1/C1** | **H2/C2** | **H3/C3** | **H4/C4** | **H5/C5** | **H6/C6** |
| --- | --- | --- | --- | --- | --- | --- |
| β-1,4-2Ac-Man-(1→  **（M2）** | 4.85/99.11 | 5.43/71.48  Ac：2.11/172.95 | 3.97/70.00 | 3.73/76.44 | 3.52/74.72 | 3.66(3.73)/60.56 |
| β-1,4-3Ac-Man-(1→  **（M3）** | 4.78/99.54 | 4.04/70.03 | 5.03/72.82  Ac：2.08/173.05 | 3.88/76.29 | 3.44/74.59 | 3.73(3.91)/60.49 |
| β-1,4-Man-(1→  **（M）** | 4.67/99.96 | 4.03/69.98 | 3.73/71.48 | 3.72/76.37 | 3.38/74.71 | 3.65(3.83)/60.19 |
| β-t-Man-(1→  **（Mt）** | 4.74/99.92 | 4.04/70.70 | 3.68/72.82 | 3.83/68.68 | 3.46/75.14 | 3.61(3.73)/62.59 |
| β-1,4-Glc-(1→  **（G4）** | 4.44/102.49 | 3.28/72.82 | 3.61/74.72 | 3.60/78.48 | 3.50/75.12 | 3.73 (3.91)/60.51 |
| β-1,4,6-Man-(1→  **（M46）** | 4.71/99.16 | 4.00/72.80 | 3.65/71.39 | 3.68/78.23 | 3.61/75.73 | 3.49(3.57)/66.61 |

**
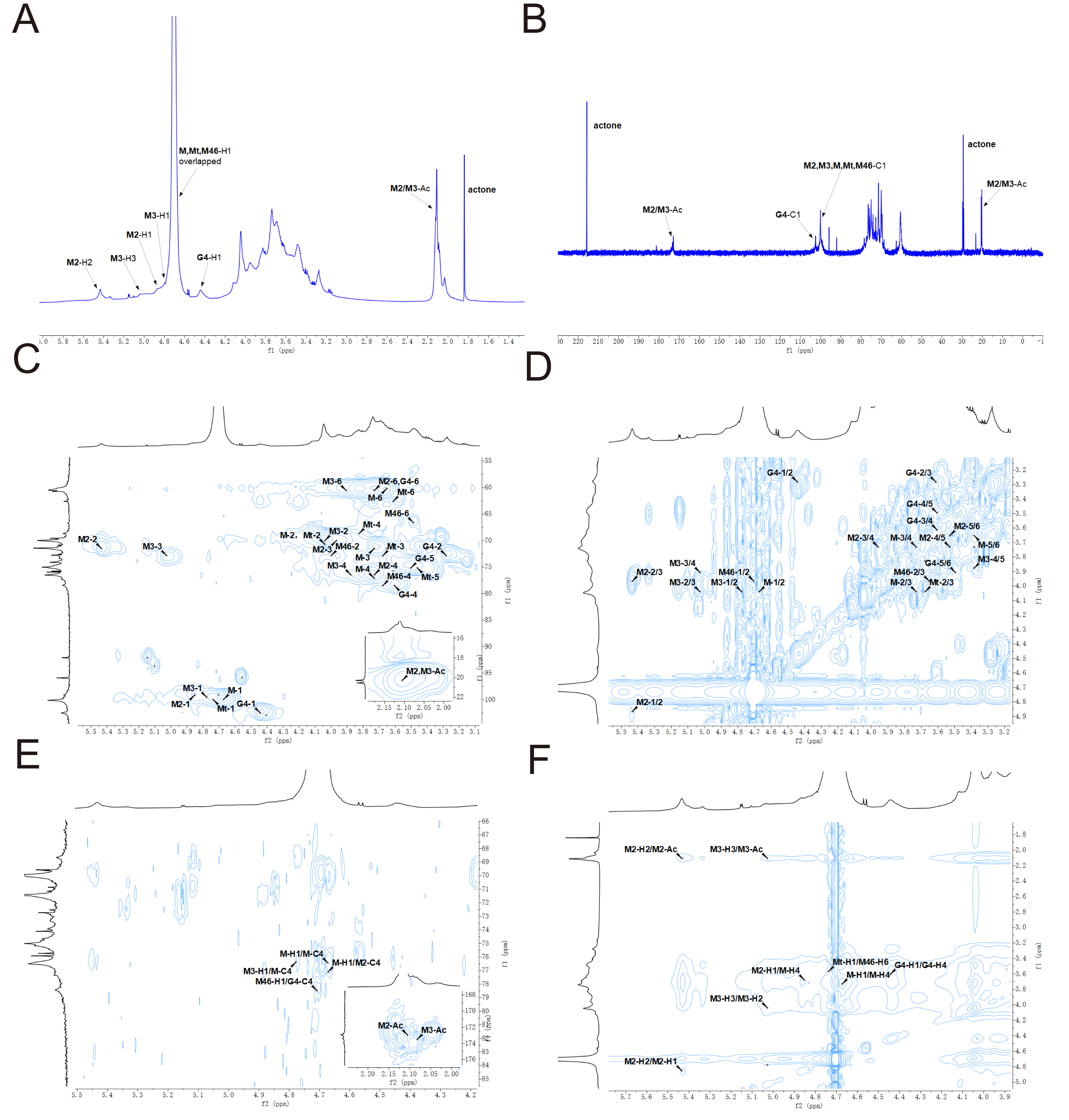
**

**Figure S2. Nuclear magnetic resonance (NMR) analysis of *Dendrobium officinale* polysaccharide (DOP).** (A) Hydrogen spectrum; (B) Carbon spectrum; (C) HSQC 2D NMR spectrum; (D) H-HCOSY 2D NMR spectrum; (E) HMBC 2D NMR spectrum; (F) NOESY spectrum.

**
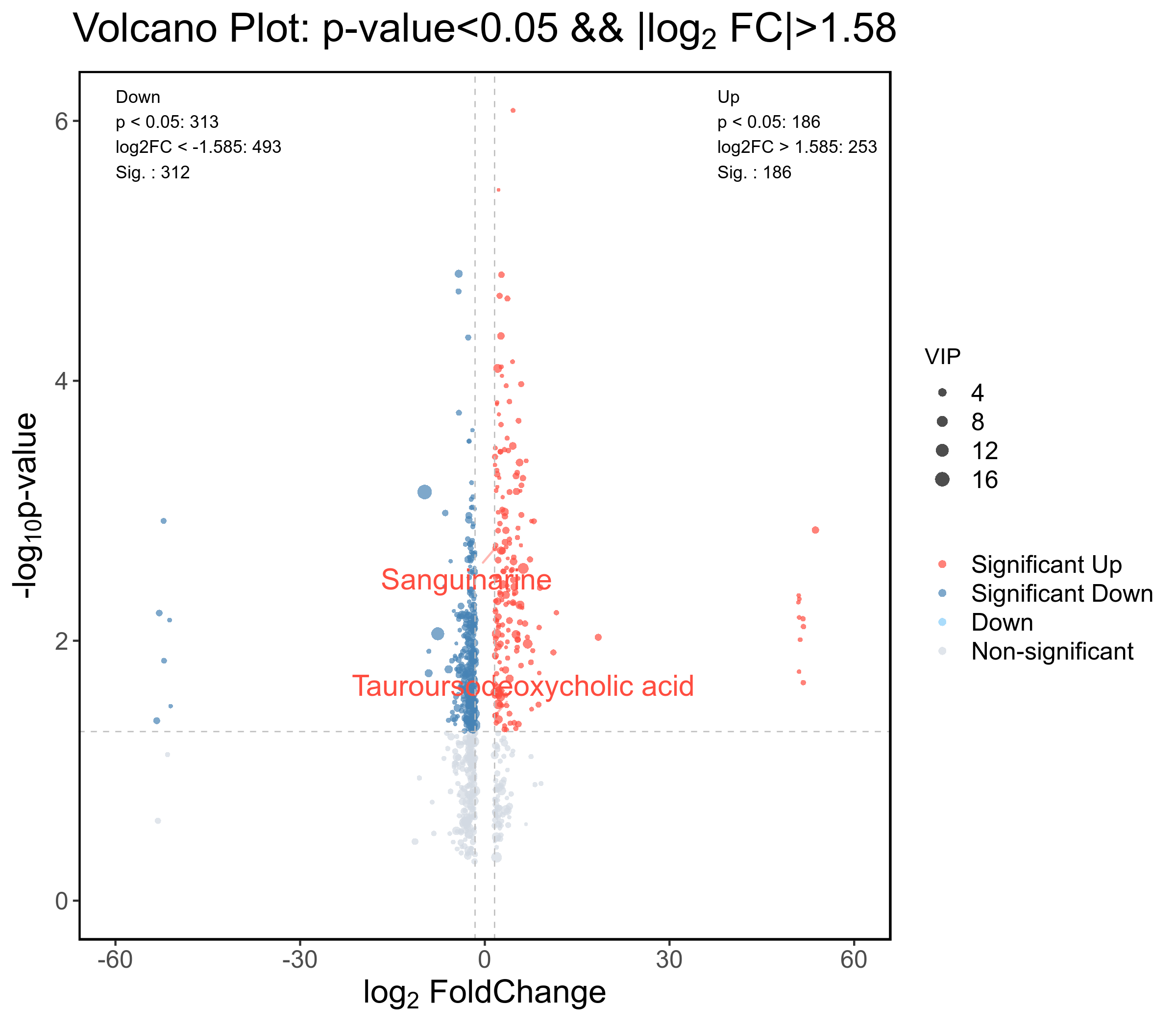
**

**Figure S3.** Volcano plot analysis based on LC-MS data showed that after DOP intervention, a total of 186 plasma metabolites were significantly upregulated, and 312 metabolites were significantly downregulated (|log2FC| > 1.58 and *p* < 0.05). To further explore its mechanism, we used the HERB database to predict the potential targets of the upregulated metabolites.


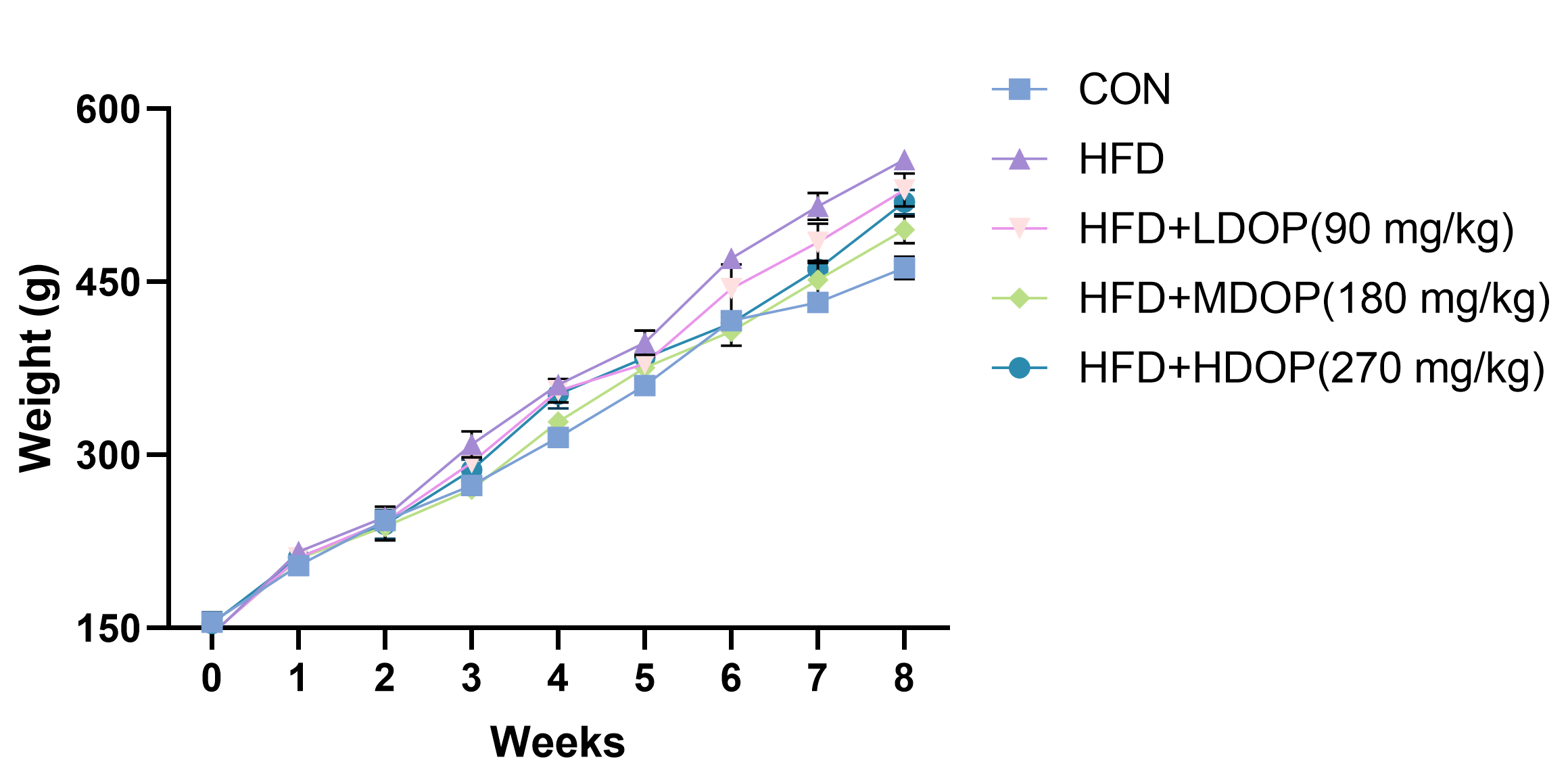


**Figure S4. The effect of different concentrations of Dendrobium officinale polysaccharide (DOP) on body weight was observed in high-fat diet (HFD)-induced rats.** Based on these preliminary results, a medium dose of 180 mg/kg (MDOP) was selected for use in subsequent experiments.
